# Case Report: Progressive myoclonus epilepsy as an early manifestation of neuronopathic Gaucher disease

**DOI:** 10.3389/fnins.2026.1742318

**Published:** 2026-01-27

**Authors:** Zhou Fang, Xixi Sun, Ying Hu, Chengjuan Xie, Xingui Chen, Yubao Jiang

**Affiliations:** 1 Department of Neurology, The First Affiliated Hospital of Anhui Medical University, Hefei, China; 2 Anhui Province Key Laboratory of Cognition and Neuropsychiatric Disorders, Hefei, China; 3 Department of Neurology, Yuexi County Hospital, Anqing, China

**Keywords:** case report, drug-resistant epilepsy, GBA1 variant, neuronopathic Gaucher disease, progressive myoclonus epilepsy

## Abstract

Gaucher disease (GD) is a lysosomal storage disorder caused by biallelic GBA1 variants. Epilepsy is uncommon in GD and rarely manifests as progressive myoclonus epilepsy (PME), making early recognition difficult. We describe a 20-year-old man with childhood-onset myoclonus that progressed to drug-resistant generalized seizures and cognitive decline. Video-electroencephalography (VEEG) showed generalized polyspike-wave discharges associated with myoclonic jerks, whereas brain magnetic resonance imaging was initially normal. Cerebrospinal fluid studies, metabolic screening, and autoimmune encephalitis antibody panels were unremarkable. Glucocerebrosidase activity was markedly reduced, and a targeted myoclonic-epilepsy gene panel identified two GBA1 variants: c.907C > A (p. Leu303Ile) and c.1505G > A (p. Arg502His), indicating a presumed compound-heterozygous state consistent with neuronopathic GD type 3. No hepatosplenomegaly or skeletal abnormalities were detected. Seizure control remained poor despite multiple antiseizure medications and vagus nerve stimulation (VNS). To contextualize this case, we systematically reviewed 22 publications encompassing 71 GD3-PME patients. Most cases presented in childhood, frequently showed typical electrophysiological patterns of generalized or multifocal polyspike-wave discharges, and had early normal MRI followed by later cerebellar or brainstem atrophy. Recurrent compound-heterozygous GBA1 variants, markedly reduced enzyme activity, and poor therapeutic response were common findings. The accompanying systematic review highlights the heterogeneity and therapeutic limitations of GD3-associated PME and underscores the importance of incorporating metabolic and genetic testing into the evaluation of unexplained PME for timely diagnosis and tailored management.

## Introduction

Gaucher disease (GD) is an autosomal-recessive lysosomal storage disorder caused by pathogenic variants in the GBA1 gene, resulting in deficient glucocerebrosidase activity and the lysosomal accumulation of glucosylceramide within macrophages (Ginzburg et al., 2004). These lipid-laden “Gaucher cells” infiltrate visceral organs such as the liver, spleen, and bone marrow, producing the classical systemic features of the disease (Beutler, 1995). Clinically, GD is categorized into three subtypes depending on neurological involvement: type 1 (non-neuronopathic), type 2 (acute neuronopathic), and type 3 (chronic neuronopathic) (Grabowski, 2008; Mistry et al., 2017). In addition, heterozygous GBA1 variants are also a major genetic risk factor for Parkinson’s disease and dementia with Lewy bodies (Nalls et al., 2013; Gan-Or et al., 2009). While types 2 and 3 are characterized by varying degrees of central nervous system (CNS) involvement, epilepsy remains an uncommon but increasingly recognized feature of GD3 (Hertz et al., 2024).

Progressive myoclonus epilepsy (PME), defined by myoclonus, generalized tonic–clonic seizures (GTCS), and progressive neurological and cognitive decline, has been rarely described in GD3 (Berkovic et al., 1986; Marseille Consensus Group, 1990; Sanz and Serratosa, 2020). Its presentation can precede or occur without visceral signs such as hepatosplenomegaly or skeletal abnormalities, often leading to diagnostic delay or misclassification as genetic generalized or mitochondrial epilepsies. Early-stage brain magnetic resonance imaging (MRI) is usually unremarkable, and seizures are frequently drug-resistant. Enzyme replacement therapy (ERT) effectively ameliorates systemic symptoms but has limited neurological benefit due to poor blood–brain barrier (BBB) penetration, whereas the efficacy of substrate reduction therapy (SRT) remains uncertain (Stirnemann et al., 2017).

Previous studies, such as that by Park et al., identified recurrent GBA1 alleles (e.g., N188S, G377S, V394L) associated with PME phenotypes, suggesting a distinct neuronopathic subtype of GD3 (Park et al., 2003). However, most available reports have focused on individual cases or small series, providing limited comparative insight into the electroclinical, genetic, and therapeutic features of GD3-associated PME.

To address this gap, we describe a young man whose PME represented the initial and isolated neurological manifestation of neuronopathic GD3 and supplement this with a structured descriptive synthesis of previously reported GD3-PME cases to provide clinical and genetic context.

## Case presentation

A 20-year-old man presented with a 10-year history of epilepsy. Early psychomotor development was normal, with no perinatal complications or febrile seizures. Myoclonic jerks first appeared at age 10, predominantly during sleep and mainly involving the upper limbs, accompanied by sudden arousal and transient postural changes. Over subsequent years, the episodes gradually evolved into GTCS that became increasingly frequent and refractory despite multiple antiseizure medications (ASMs).

As seizures generalized, cognitive performance progressively declined, characterized by reduced speech output, increasing fatigue, and noticeable impairment in daily functioning. At this stage, the Mini-Mental State Examination (MMSE) score was 15/30, indicating moderate cognitive dysfunction. On neurological examination, the patient was alert and oriented, with mild dysarthria, slightly increased muscle tone in all limbs, and symmetrical deep tendon reflexes without pathological signs. Cranial nerves were intact: pupils were equal and reactive, extraocular movements were full without gaze palsy, facial expression was symmetric, hearing was normal, and palatal and tongue movements were midline. Coordination and sensory testing were unremarkable. Gait was normal, without ataxia, spasticity, or balance impairment. Systemic examination revealed no hepatosplenomegaly, lymphadenopathy, or skeletal deformities. There were no signs of hematologic, cardiac, or respiratory disease.

The longitudinal clinical course, EEG/MRI findings, and stepwise therapeutic interventions are summarized in Table 1. This table outlines the temporal progression of seizure types, changes in ASM regimens, and corresponding clinical responses. Despite sequential polytherapy, including valproate, levetiracetam, clonazepam, lacosamide, clobazam, perampanel, and phenobarbital, seizure control remained incomplete, with only modest improvement in alertness and daytime myoclonus.

**Table 1:** Longitudinal clinical course, diagnostic evaluations, and therapeutic interventions in the patient with Gaucher disease-related progressive myoclonus epilepsy.

Time (age)	Seizure type/clinical evolution	VEEG/MRI/Other findings	Therapy	Response
Oct 2015 (11 y)	First nocturnal myoclonic seizures	Bilateral high-amplitude spike–wave complexes (frontal predominance)	VPA 250 mg QD	Controlled
Jul 2016 (12 y)	Daily GTCS	VEEG not performed; Initial MRI normal; CSF testings normal; GBA1.	VPA 250 mg BID	Frequency reduced
Oct 2017 (13 y)	Twice-daily GTCS	Generalized spike and polyspike–wave discharges	VPA 500 mg BID	Partial control
Jul 2018 (14 y)	Occasional myoclonus	Similar to prior, generalized spike–wave discharges	VPA + LEV	Intermittent myoclonus
Sep 2019 (15 y)	GTCS twice a month, stress-triggered	Right frontal-central slowing, sharp-slow discharges	VPA + LEV+OXC	1-2/month
Aug 2020 (16 y)	GTCS fourth a month	Bilateral spike-slow and polyspike-slow discharges	+CZP + LCM	Frequency decreased
Sep 2022 (18 y)	Routine follow-up, moderate control	VEEG not performed; Glucocerebrosidase activity markedly reduced; MMSE 15/30.	VPA + LEV+CZP + LCM	Frequency decreased
May 2022 (18 y)	Daily nocturnal GTCS, visual aura	VEEG not performed	Polytherapy (VPA + LEV+CZP + LCM)	Poor control
Jul 2024 (20 y)	Frequent myoclonus	Slow alpha background, bilateral polyspike-slow discharges; Follow-up MRI (outside hospital) reported cerebral atrophy.	+PER + PB + VNS	Marked improvement; MMSE 15/30

CZP, clonazepam; GTCS, generalized tonic–clonic seizure; LCM, lacosamide; LEV, levetiracetam; MMSE, Mini-Mental State Examination; MRI, magnetic resonance imaging; OXC, oxcarbazepine; PB, phenobarbital; PER, perampanel; VEEG, video-electroencephalography; VNS, vagus nerve stimulation; VPA, valproate.

**Figure 1:**
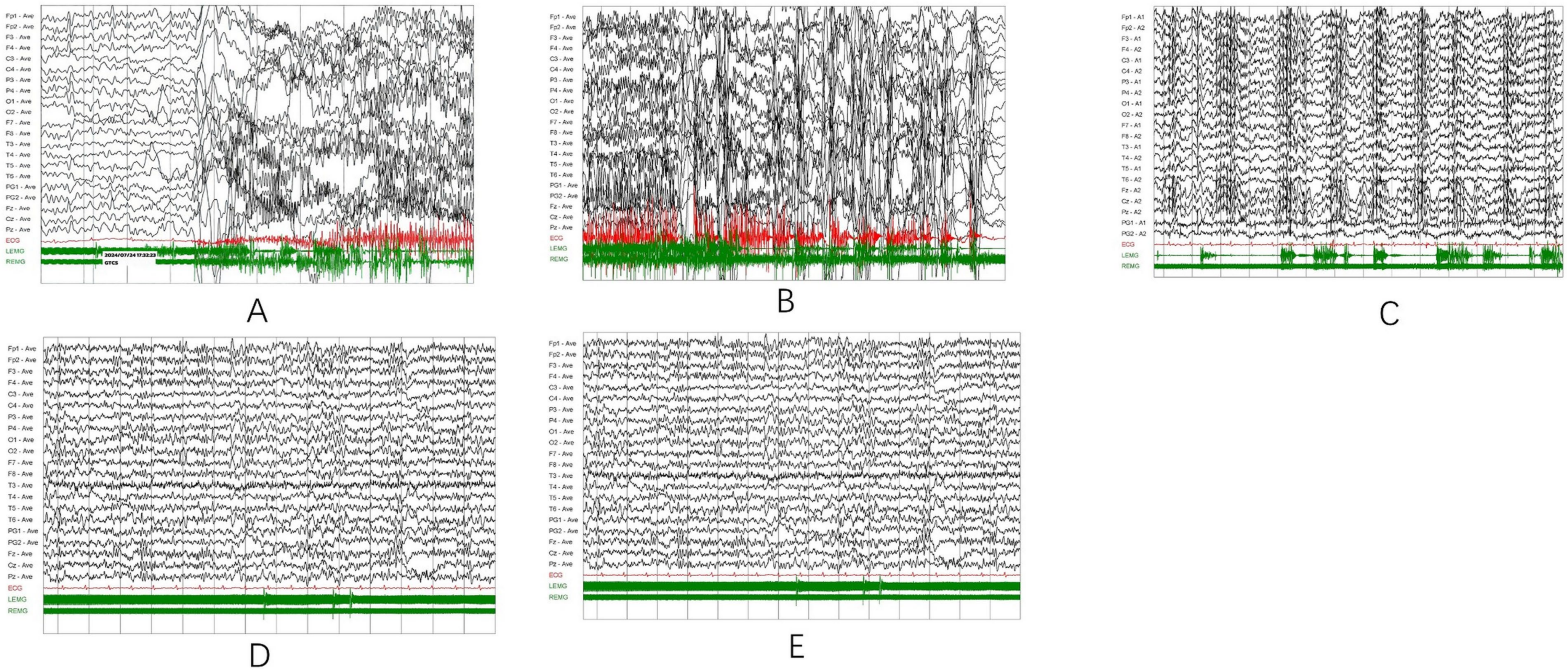
Representative ictal and interictal EEG findings in the patient at age 19. **(A–C)** Ictal EEG recordings during myoclonic seizures, showing generalized spike–wave or polyspike–wave discharges on EEG accompanied by simultaneous surface EMG bursts (green channels). The close temporal association between EEG discharges and EMG activity is consistent with cortical myoclonus involving the upper limbs and trunk. **(D,E)** Interictal video-EEG demonstrating diffuse background slowing with frequent generalized polyspike–wave discharges. EEG and surface EMG were recorded simultaneously using a 19-channel EEG system with additional EMG channels. Recording parameters: sensitivity 10 μV/mm; low-frequency filter 1 Hz; high-frequency filter 16 Hz; notch filter 50 Hz; time base 10 s/div; paper speed 23.5 mm/s. EEG, electroencephalogram; EMG, electromyography; VEEG, video-electroencephalography.

Twenty-four-hour VEEG at age 19 recorded frequent myoclonic seizures associated with generalized polyspike-wave discharges and synchronous electromyographic bursts, confirming cortical myoclonus. GTCS showed evolution from high-amplitude slow waves in the tonic phase to irregular high-frequency activity in the clonic phase. Interictal recordings demonstrated diffuse background slowing with multifocal polyspike-wave discharges, consistent with progressive myoclonus epilepsy (Figure 1).

**Figure 2:**
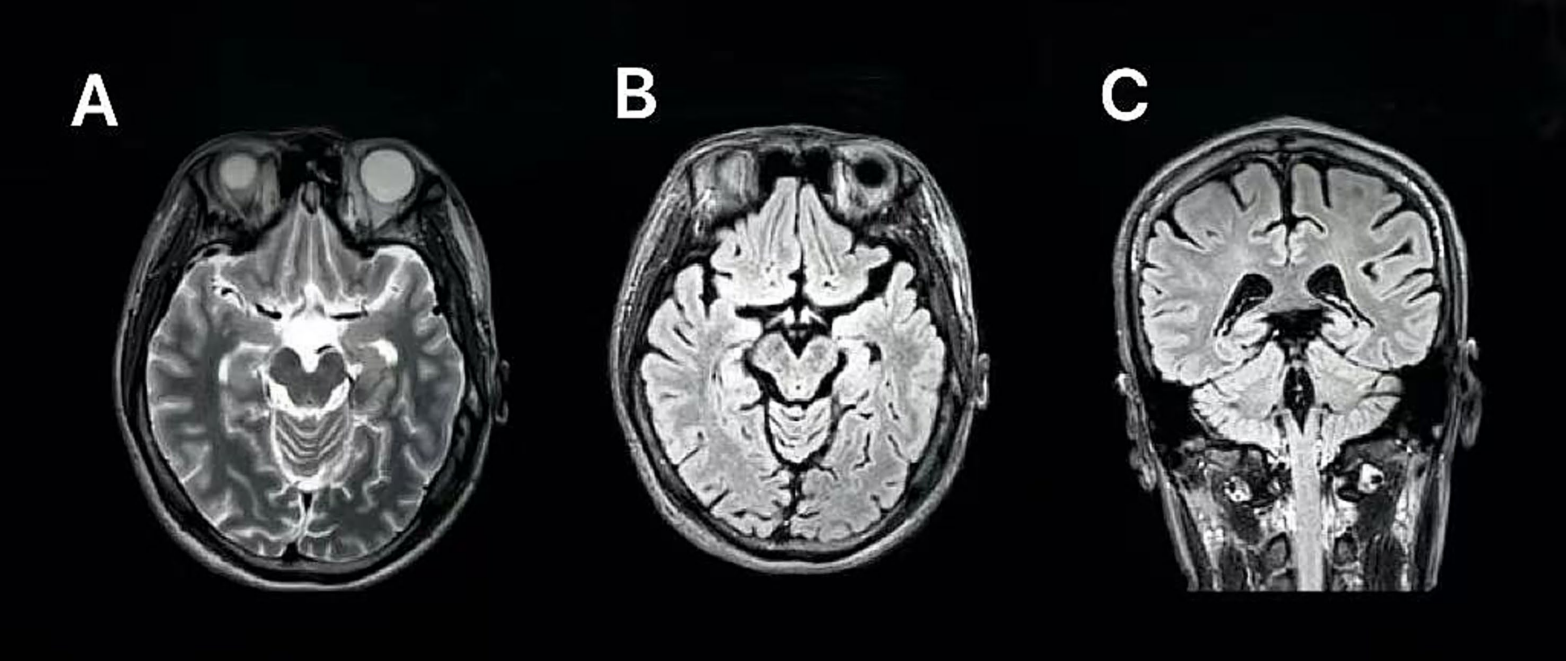
Brain MRI (3.0T) obtained during the patient’s first hospitalization in 2020. **(A)** Axial T2-weighted sequence. **(B)** Axial FLAIR sequence. **(C)** Coronal FLAIR sequence. No definite focal lesions, cortical or subcortical signal abnormalities, or cerebellar or brainstem atrophy were identified. This figure illustrates a structurally normal brain MRI at disease onset, consistent with early-stage neuronopathic Gaucher disease. MRI, magnetic resonance imaging; FLAIR, fluid-attenuated inversion recovery.

Brain magnetic resonance imaging (3.0T, T1-, T2-), fluid-attenuated inversion recovery (FLAIR)-, and diffusion-weighted imaging (DWI) sequences revealed no structural abnormalities (Figure 2). Cerebrospinal fluid (CSF) analyses and autoimmune or paraneoplastic antibody panels were unremarkable. Metabolic testing revealed a transient elevation of serum lactate (>12 mmol/L) and mild hypoglycemia (2.76 mmol/L), whereas ammonia, thyroid hormones, homocysteine, and vitamin levels were within normal limits. The transient lactate rise suggested episodic metabolic stress rather than persistent mitochondrial dysfunction.

Given the refractory epilepsy and unremarkable neuroimaging, enzymatic and genetic evaluations were pursued. Glucocerebrosidase activity was markedly reduced to 0.76 U/L (reference 1.19 to 22.23 U/L), confirming severe enzyme deficiency. Targeted sequencing identified two heterozygous GBA1 variants: c.907C > A (p. Leu303Ile, maternally inherited) and c.1505G > A (p. Arg502His, apparently *de novo*). According to ClinVar and recent clinical reports (Richards et al., 2015), c.1505G > A (p. Arg502His) is a recurrent pathogenic GBA1 variant observed in neuronopathic Gaucher disease, including type 2 and type 3, often in homozygous or compound-heterozygous states with markedly reduced enzyme activity. The c.907C > A (p. Leu303Ile) variant has more limited but emerging evidence, having been reported in patients with biochemically confirmed Gaucher disease and supported by *in silico* predictions and ACMG-based assessments as likely pathogenic. In our patient, the combination of these variants in *trans*, together with severe glucocerebrosidase deficiency and a compatible clinical phenotype, is consistent with an autosomal-recessive GBA1-related disorder, although the precise contribution of each allele to the PME-dominant presentation remains uncertain.

Despite partial symptomatic benefit from vagus nerve stimulation (VNS), which reduced seizure frequency and prevented further episodes of status epilepticus, the patient’s cognitive and motor decline continued. Neither enzyme replacement therapy (ERT) nor substrate reduction therapy (SRT) was initiated because of cost and limited accessibility. At the six-month follow-up, the patient remained ambulatory and communicative but continued to experience nocturnal seizures and progressive cognitive deterioration. His overall clinical trajectory and limited therapeutic response closely mirrored previously reported cases of GD3-associated PME in which conventional ASMs and neuromodulation provided transient benefit without halting neurodegeneration.

To place this case in context, we conducted a systematic review of 22 published reports encompassing 71 patients with GD3-associated PME. Clinical, electrophysiological, genetic, and therapeutic data were extracted and synthesized (Supplementary Tables S1, S2), allowing comparison of our patient’s presentation and outcomes with the broader literature.

## Discussion

This case describes a 20-year-old man who developed PME marked by drug-resistant seizures and cognitive decline. PME encompasses a heterogeneous group of inherited disorders characterized by myoclonus, generalized epilepsy, and progressive neurological deterioration. The principal differential diagnoses include neuronal ceroid lipofuscinosis (NCL), Unverricht-Lundborg disease (ULD), Lafora disease, sialidosis, myoclonic epilepsy with ragged-red fibers (MERRF), dentatorubral-pallidoluysian atrophy (DRPLA), and neuronopathic Gaucher disease (GD3) (Saleh et al., 2024; Orsini et al., 2019).

A detailed evaluation helped to narrow the diagnosis. The absence of visual loss, retinal degeneration, and cherry-red macula excluded NCL and sialidosis. ULD was considered unlikely since CSTB-related PME rarely leads to cognitive impairment. Lafora disease was discounted because of the absence of occipital seizures, rapid decline, or pathogenic EPM2A/EPM2B variants. MERRF was excluded based on normal muscle histology and negative mitochondrial DNA testing, and DRPLA was ruled out by negative family history. The markedly reduced glucocerebrosidase activity and compound heterozygous GBA1 variants confirmed neuronopathic GD.

Notably, this patient exhibited none of the classical systemic manifestations of GD3, such as horizontal gaze palsy, hepatosplenomegaly, or skeletal abnormalities, illustrating the wide phenotypic spectrum of GBA1-related disease (Grabowski, 2008; Hershkop et al., 2021). Similar to previously reported “neurologic-dominant” GD3 cases, PME may represent an early or even isolated presentation, which can obscure the metabolic etiology and delay diagnosis.

Epilepsy in GD is uncommon but increasingly recognized in type 3 disease (Sidransky, 2004). Reported seizure types range from nocturnal myoclonus to focal seizure or GTCS, often accompanied by cognitive and behavioral regression (McNeill et al., 2012). The electroclinical pattern in this case, characterized by sleep-related myoclonus and diffuse polyspike-wave discharges, is consistent with PME secondary to GD3. The transient lactate elevation observed in this patient suggests a contribution of mitochondrial dysfunction to epileptogenesis, in line with the hypothesis that lysosomal dysfunction, glycolipid accumulation, and chronic microglial activation collectively increase cortical excitability (Pitcairn et al., 2019; Deneubourg et al., 2022).

To contextualize this case, we conducted a structured literature search and descriptive synthesis of previously reported GD3-PME cases. Searches were performed in PubMed, Embase, and Web of Science from database inception through September 2025 using the terms (“Gaucher disease” OR “GBA1”) AND (“epilepsy” OR “progressive myoclonus epilepsy” OR “seizure”). A total of 88 records were identified, of which 22 publications met the inclusion criteria of (1) genetically or enzymatically confirmed GD type 3, (2) documented epilepsy or progressive myoclonus epilepsy, and (3) availability of patient-level clinical information. Reviews, editorials, non-human studies, and reports lacking individual data were excluded. Potential duplicate or overlapping cases were identified by comparing genotype, age, sex, and clinical features; when overlap was suspected, only the most complete report was retained.

Because nearly all eligible studies consisted of heterogeneous single-case reports or small case series with inconsistently reported variables, findings were synthesized descriptively rather than through a PRISMA-based systematic review or meta-analysis. Missing data were coded as “not reported,” and no formal study quality appraisal tool was applied for the same reason, although each case was independently reviewed for internal consistency and clarity of diagnosis. This descriptive approach is aligned with prior broader reviews of neurological involvement in Gaucher disease, including the recent systematic review by Imbalzano et al. (2024), which highlighted the predominance of case-based evidence and the wide heterogeneity of neurological phenotypes. In contrast to that broader work, the present synthesis focuses specifically on the GD3-PME phenotype, providing a consolidated overview of 71 published cases to facilitate comparison with the current patient.

**Figure 3:**
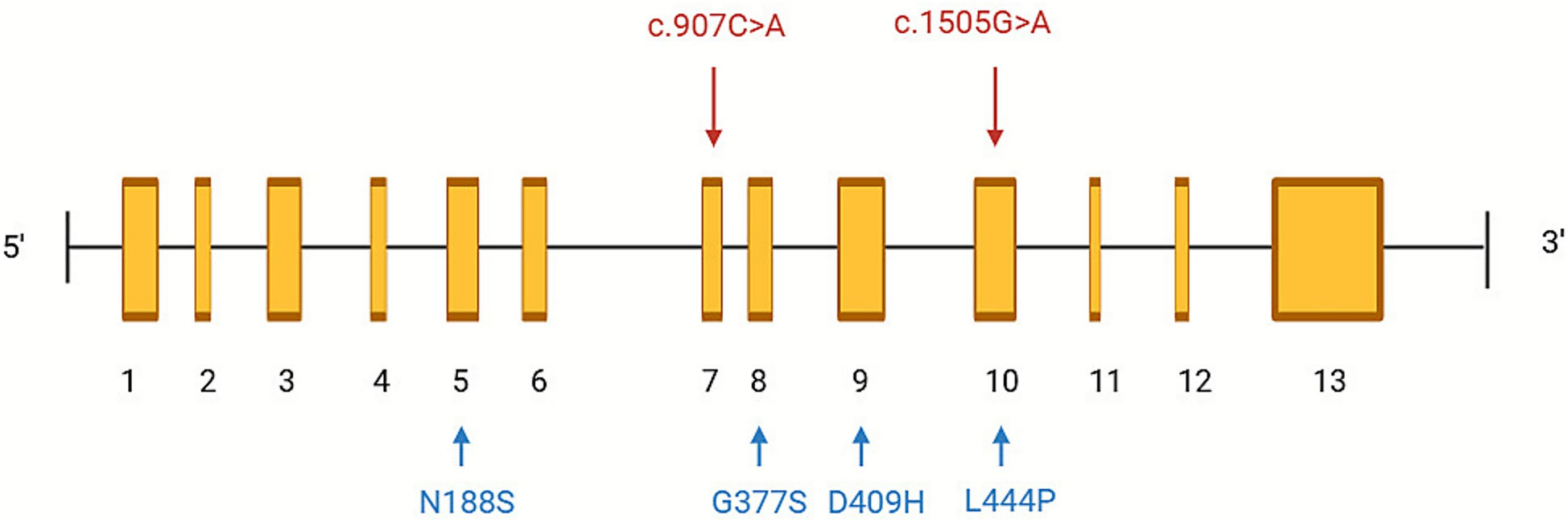
Schematic representation of the *GBA1* gene and distribution of pathogenic variants associated with neuronopathic Gaucher disease and progressive myoclonus epilepsy. The *GBA1* gene consists of 13 exons arranged from the 5′ to 3′ direction. The two variants identified in the present patient, c.907C > A (p. Leu303Ile, exon 7; maternally inherited) and c.1505G > A (p. Arg502His, exon 10; apparently *de novo*), are highlighted in red. Previously reported recurrent pathogenic or likely pathogenic *GBA1* variants associated with neuronopathic Gaucher disease and GD3-related progressive myoclonus epilepsy, including N188S (exon 5), L444P (exon 8), D409H (exon 9), and G377S (exon 10), are shown for comparison. This schematic places the patient’s variants within the broader mutational spectrum of GD3-associated PME and illustrates the compound-heterozygous configuration and marked genetic heterogeneity underlying this phenotype. Abbreviations: GD, Gaucher disease; PME, progressive myoclonus epilepsy.

To contextualize our findings, we systematically reviewed 22 previous reports encompassing 71 patients with GD3-associated PME (Supplementary Tables S1, S2) (Park et al., 2003; Kraoua et al., 2011; Biegstraaten et al., 2011; Narita et al., 2016; Lee et al., 2012; Filocamo et al., 2004; Poffenberger et al., 2020; Charkhand et al., 2019; Yang et al., 2024; Serrão et al., 2024; Tajima et al., 2010; Niu et al., 2022; Oguri et al., 2020; Ciana et al., 2020; Yamaguchi-Takegami et al., 2024; Tahara et al., 2022; Capablo et al., 2007; Sestito et al., 2017; Kim et al., 2020; Tonin et al., 2019; Miyahara et al., 2009; Seeman et al., 1996). A visual summary is available in the Supplementary Figures 1A–G. Because the included publications were mainly case reports and small case series, the available clinical variables, follow-up duration, and diagnostic approaches were heterogeneous. The majority of cases presented during childhood or adolescence, with a median onset age of approximately 9 years. Approximately two-thirds showed hepatosplenomegaly or hematologic abnormalities, whereas one-third manifested primarily neurological symptoms without visceral involvement, supporting PME as an early or isolated phenotype within the neuronopathic GD spectrum. EEG findings were consistent across studies, typically revealing generalized or multifocal polyspike-wave discharges with photoparoxysmal or sleep activation. MRI findings were often normal at onset but later demonstrated cerebellar or brainstem atrophy, suggesting that electrophysiological changes precede structural degeneration. The most recurrent GBA1 variants included N188S, L444P, D409H, G377S, and RecNciI, all associated with markedly reduced enzyme activity. For clarity, a schematic representation of the GBA1 gene indicating the patient’s variants alongside commonly reported pathogenic mutations has been incorporated (Figure 3). Therapeutic outcomes were generally poor: nearly 60% of patients showed minimal seizure control despite multiple ASMs and ERT, while only 19/71 achieved ≥ 50% seizure reduction with substrate reduction or chaperone therapy, and seizure freedom was rare (4/71). Overall seizure control was poor, with limited response to ASMs, neuromodulation, or ERT, although a minority of patients achieved partial improvement with substrate reduction or chaperone therapy. Our case closely mirrors these observations. The patient exhibited drug-resistant PME with diffuse polyspike-wave discharges and cognitive decline but no visceral involvement, consistent with previously described “neurologic-dominant” GD3 phenotypes such as those reported by Park et al. (2003). These convergent findings strengthen the notion that severe neuronal vulnerability in GD can manifest independently of systemic burden, highlighting the need for brain-penetrant therapeutic approaches (Kong et al., 2022).

This patient’s clinical course and treatment response align closely with these observations. Drug-resistant PME, diffuse polyspike-wave discharges, and progressive cognitive decline occurred in the absence of systemic burden, emphasizing that severe neuronal vulnerability can occur independently of visceral pathology. These convergent findings highlight the need for therapies capable of crossing the blood–brain barrier (BBB) to target the underlying neurodegeneration.

The management of GD-associated epilepsy remains challenging. Conventional ASMs generally provide partial symptom control. Management of GD-associated epilepsy remains challenging. Conventional ASMs typically provide partial benefit, and VNS may reduce seizure frequency and prevent status epilepticus but rarely halts neurological deterioration. ERT improves systemic manifestations but fails to affect CNS involvement due to limited BBB penetration. Substrate reduction therapy (e.g., miglustat) can reach the CNS and may offer modest neurological improvement, although evidence remains inconclusive (Shimizu et al., 2023). In this case, neither ERT nor SRT was initiated due to limited access. Adjunctive strategies such as ketogenic diet and mitochondrial cofactors have been explored for symptomatic stabilization, but current data are preliminary (Jiang et al., 2022; Guerreiro et al., 2024). Emerging approaches, including pharmacological chaperones, substrate inhibitors, and gene-based therapies, offer promising avenues for future intervention.

This report has limitations. First, as this is a single-case description, the generalizability of the patient’s clinical trajectory and treatment response is inherently limited, and the findings should therefore be interpreted with caution. Second, ERT and SRT were not initiated due to financial and accessibility constraints; therefore, the potential influence of disease-specific therapy on seizure control or neurological decline could not be evaluated. Third, the literature synthesis relied primarily on heterogeneous case reports and small case series with variable diagnostic rigor and data completeness, which may introduce reporting bias and limit comparability across published cases. Finally, although two pathogenic or likely pathogenic GBA1 variants (c.907C > A and c.1505G > A) were identified in our patient, the genotype–phenotype correlation in GD, particularly in neuronopathic subtypes, remains highly variable. Phenotypic expression is shaped not only by the specific variants but also by residual enzyme activity, modifier genes, lysosomal homeostasis, and other biological or environmental factors. As a result, the precise contribution of these variants to the severity of PME in this case cannot be definitively established. Despite these limitations, this case adds to growing evidence that PME can represent an early or isolated neurological manifestation of neuronopathic GD. It underscores the diagnostic value of incorporating metabolic and genetic screening into the evaluation of unexplained PME, particularly when neuroimaging is normal. Early recognition enables timely counseling, individualized management, and close longitudinal monitoring.

## Conclusion

This case expands the recognized phenotypic spectrum of Gaucher disease by showing that progressive myoclonus epilepsy may precede or occur without systemic involvement. It reinforces the importance of metabolic and genetic testing in patients with unexplained PME and highlights the urgent need for brain-penetrant, disease-modifying therapies for neuronopathic GD.

### Patient perspective

When the patient first began to experience uncontrollable jerks and seizures, his family was confused and deeply anxious about the cause. Over the years, repeated hospital visits and changing medications offered little improvement, and the uncertainty surrounding his illness became an emotional and financial burden for them. Receiving a definitive diagnosis of neuronopathic Gaucher disease finally brought both relief and understanding. Although the treatment options remain limited, the family expressed gratitude for having an explanation that connected his symptoms and guided further care. They also hoped that sharing their experience could help other families with unexplained epilepsy receive earlier recognition and support.

## Data Availability

The original contributions presented in the study are included in the article/Supplementary material, further inquiries can be directed to the corresponding author.
