# Supplementary material for: Case Report: Progressive myoclonus epilepsy as an early manifestation of neuronopathic Gaucher disease
Source: Front Neurosci. 2026 Jan 27;20:1742318. doi: 10.3389/fnins.2026.1742318 (PMC12886469; doi:10.3389/fnins.2026.1742318)
Supplement: Supplementary file 1 [file Table_1.DOCX]

Table1. Longitudinal clinical course, diagnostic evaluations, and therapeutic interventions in the patient with Gaucher disease-related progressive myoclonus epilepsy.

| **Time (Age)** | **Seizure type /**  **clinical evolution** | **VEEG / MRI/Other findings** | **Therapy** | **Response** |
| --- | --- | --- | --- | --- |
| Oct 2015 (11 y) | First nocturnal myoclonic seizures | Bilateral high-amplitude spike-wave complexes (frontal predominance) | VPA 250 mg QD | Controlled |
| Jul 2016 (12 y) | Daily GTCS | VEEG not performed;  Initial MRI normal;  CSF testings normal;  GBA1. | VPA 250 mg BID | Frequency reduced |
| Oct 2017 (13 y) | Twice-daily GTCS | Generalized spike and polyspike-wave discharges | VPA 500 mg BID | Partial control |
| Jul 2018 (14 y) | Occasional myoclonus | Similar to prior, generalized spike-wave discharges | VPA+LEV | Intermittent myoclonus |
| \| Sep 2019(15 y) \| \| --- \| | GTCS twice a month, stress-triggered | Right frontal-central slowing, sharp-slow discharges | VPA+LEV+OXC | 1-2/month |
| Aug 2020 (16 y) | GTCS fourth a month | Bilateral spike-slow and polyspike-slow discharges | +CZP + LCM | Frequency decreased |
| Sep 2022 (18 y) | Routine follow-up, moderate control | VEEG not performed;  Glucocerebrosidase activity markedly reduced;  MMSE 15/30. | VPA+LEV+CZP+LCM | Frequency decreased |
| May 2022 (18 y) | Daily nocturnal GTCS, visual aura | VEEG not performed | Polytherapy (VPA+LEV+CZP+LCM) | Poor control |
| \| Jul 2024 (20 y) \| \| --- \| | Frequent myoclonus | Slow alpha background, bilateral polyspike-slow discharges;  Follow-up MRI (outside hospital) reported cerebral atrophy. | +PER + PB + VNS | Marked improvement; MMSE 15/30 |

Abbreviations: CZP, clonazepam; GTCS, generalized tonic-clonic seizure; LCM, lacosamide; LEV, levetiracetam; MMSE, Mini-Mental State Examination; MRI, magnetic resonance imaging; OXC, oxcarbazepine; PB, phenobarbital; PER, perampanel; VEEG, video-electroencephalography; VNS, vagus nerve stimulation; VPA, valproate.
