# Supplementary material for: Case Report: Progressive myoclonus epilepsy as an early manifestation of neuronopathic Gaucher disease
Source: Front Neurosci. 2026 Jan 27;20:1742318. doi: 10.3389/fnins.2026.1742318 (PMC12886469; doi:10.3389/fnins.2026.1742318)
Supplement: Supplementary file 2 [file Table_2.DOCX]

**Table S1.** **Clinical and electrophysiological characteristics of reported patients with Gaucher disease and progressive myoclonus epilepsy.**

| Original Source  (first author, year) | Age at Onset  (y) | | Sex | Visceral involvement  (Yes/No/Not reported） | EEG/MRI findings |
| --- | --- | --- | --- | --- | --- |
| Kraoua 2011– Patient 2 | | 12 | M | No | Not reported |
| Kraoua 2011– Patient 5 | | 12 | M | Yes | Not reported |
| Kraoua 2011 – Patient 7 | | 3 | M | Yes | Not reported |
| Biegstraaten 2011-Twin A | | 22 | F | Yes | EEG: epileptiform discharges; MRI: initially normal, later mild cerebellar atrophy. |
| Narita 2016-Patient 1 | | 28 | F | Yes | Not reported |
| Narita 2016-Patient 2 | | 20 | F | Yes | Not reported |
| Narita 2016-Patient 3 | | 15 | F | Yes | Not reported |
| Narita 2016-Patient 5 | | 25 | F | Yes | Not reported |
| Lee 2012-Patient 14 | | 2.8 | M | Yes | Not reported |
| Lee 2012-Patient 16 | | 15.8 | F | Yes | Not reported |
| Lee 2012-Patient 17 | | 11.9 | F | Yes | Not reported |
| Lee 2012-Patient 19 | | 3.3 | F | Yes | Not reported |
| Filocamo 2004 | | 11 | M | Yes | EEG: generalized/multifocal discharges with photoparoxysmal response; MRI/CT: normal. |
| Poffenberger 2020-Patient 1 | | 8 | M | Not reported | Not reported |
| Poffenberger 2020-Patient 2 | | 6 | F | Not reported | Not reported |
| Poffenberger 2020-Patient 5 | | 29 | F | Not reported | Not reported |
| Poffenberger 2020-Patient 16 | | 5 | M | Not reported | Not reported |
| Poffenberger 2020-Patient 18 | | 8 | M | Not reported | Not reported |
| Poffenberger 2020-Patient 39 | | 7 | M | Not reported | Not reported |
| Poffenberger 2020-Patient 45 | | 19 | M | Not reported | Not reported |
| Poffenberger 2020-Patient 47 | | 20 | M | Not reported | Not reported |
| Poffenberger 2020-Patient 54 | | 28 | M | Not reported | Not reported |
| Poffenberger 2020-Patient 56 | | 11 | M | Not reported | Not reported |
| Poffenberger 2020-Patient 66 | | 6 | M | Not reported | Not reported |
| Charkhand 2019-Patient 1 | | 15 | M | Not reported | EEG: multifocal/generalized discharges with photosensitivity; MRI: normal. |
| Charkhand 2019-Patient 2 | | 21 | F | Not reported | Not reported |
| Yang 2024-Patient 1 | | 13.6 | Not reported | Not reported | EEG: polyspike–wave discharges; MRI: not reported. |
| Yang 2024-Patient 2 | | 8.3 | Not reported | Not reported | EEG: polyspike–wave discharges with ESES pattern; MRI: not reported. |
| Yang 2024-Patient 3 | | 16.9 | Not reported | Not reported | EEG: polyspike–wave discharges; MRI: not reported. |
| Yang 2024-Patient 4 | | 19.8 | Not reported | Not reported | EEG: polyspike–wave discharges; MRI: not reported. |
| Yang 2024-Patient 6 | | 5.2 | Not reported | Not reported | EEG: spike–wave discharges; MRI: not reported. |
| Yang 2024-Patient 7 | | 20.2 | Not reported | Not reported | EEG: polyspike–wave discharges; MRI: not reported. |
| Yang 2024-Patient 8 | | 7.8 | Not reported | Not reported | EEG: spike–wave discharges; MRI: not reported. |
| Yang 2024-Patient 9 | | 8.9 | Not reported | Not reported | EEG: sharp– and spike–wave discharges; MRI: not reported. |
| Yang 2024-Patient 11 | | 6 | Not reported | Not reported | EEG: polyspike–wave discharges; MRI: not reported. |
| Serrãol 2024 | | 43 | F | Not reported | EEG: generalized spikes, polyspikes, and polyspike–wave discharges (8–20 Hz, maximal over parietocentral regions); MRI: not reported. |
| Tajima 2010 | | 16 | F | Yes | EEG: diffuse polyspike bursts superimposed on alpha activity; MRI: normal. |
| Niu 2022-2Patient 7 | | Not reported | M | Not reported | EEG: abnormal background with diffuse fast activity; MRI: white-matter dysplasia and progressive parenchymal atrophy. |
| Oguri 2019-Patient 1 | | 11.4 | F | Yes | EEG: 3–4 Hz spike–wave discharges; MRI: mild cerebral and cerebellar atrophy. |
| Oguri 2019-Patient 2 | | 8 | F | No | EEG: polyspike–wave discharges; MRI: mild cerebral and cerebellar atrophy. |
| Oguri 2019-Patient 3 | | 7 | F | No | EEG: polyspike–wave discharges; MRI: mild cerebral and cerebellar atrophy. |
| Ciana 2020- Patient 1 | | 6 | F | Yes | EEG: initially normal, later showing generalized epileptiform discharges with increasing frequency; MRI: normal. |
| Ciana 2020- Patient 2 | | 14 | M | Yes | Not reported |
| Yamaguchi-Takegami 2023 | | 50 | F | Yes | EEG: 3 Hz spike–slow-wave complexes induced by photic stimulation with central–parietal paroxysms; MRI: lacunar infarction in the right putamen and T2 hyperintensities in periventricular, deep, and subcortical white matter with frontal and temporal atrophy. |
| Tahara 2022 | | 27 | F | Yes | EEG: mild spiky activity during photic stimulation; MRI: normal. |
| Park 2003-Patient 1 | | 5 | M | Yes | EEG: not specified; MRI: normal. |
| Park 2003-Patient 2 | | 4 | F | Yes | EEG: multifocal seizure disorder with slow background activity; MRI: not reported. |
| Park 2003-Patient 3 | | 5 | F | Yes | Not reported |
| Park 2003-Patient 4 | | 2 | M | Yes | EEG: epileptiform discharges with slow background activity; MRI: normal. |
| Park 2003-Patient 5 | | 10 | M | Yes | EEG: epileptiform discharges with slow background activity; MRI: normal. |
| Park 2003-Patient 6 | | 1 | M | Yes | EEG: photoparoxysmal response; MRI: not specified. |
| Park 2003-Patient 7 | | 3 | M | Yes | EEG:not reported;MRI: normal. |
| Park 2003-Patient 8 | | 2 | M | Yes | EEG: diffuse irregular background with multifocal independent spikes; MRI: not reported. |
| Park 2003-Patient 9 | | 2 | F | Yes | EEG: slow background with left occipital spikes; MRI: normal. |
| Park 2003-Patient 10 | | 14 | M | Yes | EEG: frequent biphasic or polyphasic spikes with slow background; MRI: normal. |
| Park 2003-Patient 11 | | 7 | M | Yes | EEG: slow background with spike-wave and polyspike discharges showing photosensitivity; MRI: cerebellar and brainstem atrophy. |
| Park 2003-Patient 12 | | 14 | M | Yes | EEG: paroxysmal rapid spikes with slow background; MRI: normal. |
| Park 2003-Patient 13 | | 22 | F | Yes | EEG:not reported;MRI: normal. |
| Park 2003-Patient 14 | | 4 | F | Yes | EEG: complex partial seizure activity; MRI: basal ganglia atrophy with old lacunar lesions. |
| Park 2003-Patient 15 | | 38 | F | Yes | EEG: partial seizure activity; MRI: not reported. |
| Park 2003-Patient 16 | | 4 | M | Yes | EEG: discharges suggestive of petit-mal myoclonic seizure pattern; MRI: not reported. |
| Capablo 2007 | | 31 | M | Yes | EEG: slow background with generalized 4–5 Hz fast spike–wave discharges and left temporal epileptiform activity spreading contralaterally; no photoparoxysmal response observed. MRI: not reported. |
| Sestito 2017-Patient 1 | | 1.2 | M | Yes | Not reported |
| Sestito 2017-Patient 3 | | 0.7 | F | Yes | Not reported |
| Kim 2020-Patient 1 | | 20.1 | F | Yes | EEG: not reported; MRI: stable spectroscopy findings (no significant changes in NAA/Cr or Cho/Cr ratios). |
| Kim 2020-Patient 2 | | 17.3 | F | Yes | EEG: not reported; MRI: stable spectroscopy findings (no significant changes in NAA/Cr or Cho/Cr ratios). |
| Kim 2020-Patient 3 | | 14.6 | F | Yes | EEG: not reported; MRI: stable spectroscopy findings (no significant changes in NAA/Cr or Cho/Cr ratios). |
| Kim 2020-Patient 4 | | 17.2 | F | Yes | EEG: not reported; MRI: stable spectroscopy findings (no significant changes in NAA/Cr or Cho/Cr ratios). |
| Tonin 2018 | | 11 | F | Yes | EEG: frequent generalized and multifocal polyspike–wave discharges with ictal eyelid myoclonia and absences; prominent photosensitivity and photoparoxysmal response during photic stimulation; severe activation of paroxysmal discharges during sleep. MRI: not reported. |
| Miyahara 2009-Patient 4 | | 8 | F | Yes | EEG: generalized slow background with diffuse spike/polyspike–wave discharges, repetitive diffuse or focal spikes, and frontal theta bursts (centroparietal predominance). MRI: not reported. |
| Seeman 1996 | | 7 | M | Yes | EEG: generalized 3–4 Hz high-voltage spike–wave discharges with frontal predominance, accompanying brief generalized myoclonus of trunk and limbs. MRI: not reported. |

**Abbreviations:** EEG, electroencephalography; MRI, magnetic resonance imaging; F, female; M, male.

**Table S2. Genetic and biochemical profiles of reported patients with Gaucher disease and progressive myoclonus epilepsy.**

| **Original Source**  **(first author, year)** | **Genotype** | **Enzyme activity**  **(GCase U/L or relative)** | **Interventions (ASMs, ERT, SRT, Ambroxol, etc.)** | **Seizure Outcome** |
| --- | --- | --- | --- | --- |
| Kraoua 2011 – Patient 2 | D409H/L444P | Not reported | ERT+SRT | No response |
| Kraoua 2011 – Patient 5 | RecNcil/- | Not reported | ERT | No response |
| Kraoua 2011 – Patient 7 | RecNcil/V394L | Not reported | ERT | No response |
| Biegstraaten 2011-Twin A | N188S/N188S | 1.9(↓, ref 10-20 nmol/mg/h) | ERT+ASMs | No response |
| Narita 2016-Patient 1 | N188S/G193W | Markedly reduced | ERT+ASMs+Ambroxol | ≥50% Reduction |
| Narita 2016-Patient 2 | N188S/- | Markedly reduced | ERT+ASMs+Ambroxol | ≥50% Reduction |
| Narita 2016-Patient 3 | N188S/- | Markedly reduced | ERT+ASMs+Ambroxol | ≥50% Reduction |
| Narita 2016-Patient 5 | D409H/IVS10-1 | Markedly reduced | BMT+ASMs+Ambroxol | ≥50% Reduction |
| Lee 2012-Patient 14 | L444P/F213I | Not reported | ERT | No response |
| Lee 2012-Patient 16 | N188S/R257Q | 11.6(↓, ref 20-80 pmol/min/mg) | ERT | No response |
| Lee 2012-Patient 17 | N188S/R257Q | 8.1(↓, ref 20-80 pmol/min/mg) | ERT | No response |
| Lee 2012-Patient 19 | F213I/L444P | 5.1(↓, ref 20-80 pmol/min/mg) | ERT | No response |
| Filocamo 2004 | N188S/S107L | Markedly reduced | ASMs | No response |
| Poffenberger 2020-Patient 1 | V394L/RecNcil | Not reported | ERT+ASMs | No response |
| Poffenberger 2020-Patient 2 | L444P/F213I | Not reported | ERT+ASMs | No response |
| Poffenberger 2020-Patient 5 | G377S/Y205C | Not reported | ERT+ASMs | No response |
| Poffenberger 2020-Patient 16 | D409H/RecNcil | Not reported | ERT | No response |
| Poffenberger 2020-Patient 18 | G377S/R463H | Not reported | ERT | No response |
| Poffenberger 2020-Patient 39 | N188S+Rec7/- | Not reported | ERT | No response |
| Poffenberger 2020-Patient 45 | N188S/RecNcil | Not reported | ERT+ASMs | No response |
| Poffenberger 2020-Patient 47 | K157Q/D140H+E326K | Not reported | ERT+ASMs | No response |
| Poffenberger 2020-Patient 54 | c.del55bp/F216Y | Not reported | ERT+ASMs | No response |
| Poffenberger 2020-Patient 56 | N188S/S107L | Not reported | Not reported | Not reported |
| Poffenberger 2020-Patient 66 | G377S/c.102delT | Not reported | Not reported | Not reported |
| Charkhand 2019-Patient 1 | G377S/G195E | 1.62(↓, ref 15.2 ± 6.3 nmol/h/mg) | ERT+ASMs+Ambroxol | No response |
| Charkhand 2019-Patient 2 | N188S/R463H | 0.6(↓, ref 15.2 ± 6.3 nmol/h/mg ) | ERT+ASMs+Ambroxol | ≥50% Reduction |
| Yang 2024-Patient 1 | Not reported | Not reported | ERT+ASMs | No response |
| Yang 2024-Patient 2 | Not reported | Not reported | ERT+ASMs | No response |
| Yang 2024-Patient 3 | Not reported | Not reported | ERT+ASMs | No response |
| Yang 2024-Patient 4 | Not reported | Not reported | ERT+ASMs | No response |
| Yang 2024-Patient 6 | Not reported | Not reported | ERT+ASMs | No response |
| Yang 2024-Patient 7 | Not reported | Not reported | ERT+ASMs | No response |
| Yang 2024-Patient 8 | Not reported | Not reported | ERT+ASMs | No response |
| Yang 2024-Patient 9 | Not reported | Not reported | ERT+ASMs | No response |
| Yang 2024-Patient 11 | Not reported | Not reported | ERT+ASMs | No response |
| Serrãol 2024 | D416N/D416N | Markedly reduced | Not reported | Not reported |
| Tajima 2010 | N188S / G199D | 7.4 (↓, ref 49.1±2.2 nmol/mg/h) | ASMs | No response |
| Niu 2022-Patient 7 | N227S/L483P | Markedly reduced | ASMs | No response |
| Oguri 2019-Patient 1 | N188S/IVS2+1 | Not reported | ERT+ASMs | ≥50% Reduction |
| Oguri 2019-Patient 2 | N188S/- | Not reported | ERT+ASMs | ≥50% Reduction |
| Oguri 2019-Patient 3 | N188S/- | Not reported | ERT+ASMs | ≥50% Reduction |
| Ciana 2020- Patient 1 | N188S / IVS2+1G>A | Not reported | ERT+ASMs+Ambroxol | Seizure-free |
| Ciana 2020- Patient 2 | L444P/L444P | Not reported | ERT+ASMs+Ambroxol | no response |
| Yamaguchi-Takegami 2023 | N227S / G416S | 1.3 (↓, nmol/mg/h) | ERT+ASMs | Seizure-free |
| Tahara 2022 | P252I / L483P | Markedly reduced | ERT+ASMs+Ambroxol | Seizure-free |
| Park 2003-Patient 1 | V394L/RecNciI | Markedly reduced | ERT+ASMs | No response |
| Park 2003-Patient 2 | L444P/F213I | Markedly reduced | ERT+SRT+ASMs | No response |
| Park 2003-Patient 3 | G325R/C342G | Markedly reduced | ASMs | No response |
| Park 2003-Patient 4 | I402F/E326K+G377S | Markedly reduced | ERT+BMT+ASMs | No response |
| Park 2003-Patient 5 | L444P+recomb/G202R | Markedly reduced | ERT+ASMs | No response |
| Park 2003-Patient 6 | V394L/RecNciI | Markedly reduced | ERT+ASMs | No response |
| Park 2003-Patient 7 | D409H/R131L | Markedly reduced | ASMs | No response |
| Park 2003-Patient 8 | G377S/c.1020 delT | Markedly reduced | ERT+ASMs | No response |
| Park 2003-Patient 9 | L444P/L444P | Markedly reduced | ASMs | No response |
| Park 2003-Patient 10 | K157Q/D140H+E326K | Markedly reduced | ASMs | ≥50% Reduction |
| Park 2003-Patient 11 | N188S/RecNciI | Markedly reduced | ASMs | ≥50% Reduction |
| Park 2003-Patient 12 | N188S/c.84-85insG | Markedly reduced | ASMs | ≥50% Reduction |
| Park 2003-Patient 13 | N188S/Recomb | Markedly reduced | ASMs | ≥50% Reduction |
| Park 2003-Patient 14 | G377S/Y205C | Markedly reduced | ERT+ASMs | ≥50% Reduction |
| Park 2003-Patient 15 | N188S/RecNciI | Markedly reduced | ASMs | ≥50% Reduction |
| Park 2003-Patient 16 | c.1263–1317del/ F216y | Markedly reduced | ASMs | No response |
| Capablo 2007 | L444P / (E326K + N188S) | Markedly reduced | ERT+SRT+ASMs | Seizure-free |
| Sestito 2017-Patient 1 | L444P/L444P | Markedly reduced | ERT+ASMs | No response |
| Sestito 2017-Patient 3 | L444P/L444P | Markedly reduced | ERT+ASMs | No response |
| Kim 2020-Patient 1 | N227S / R296Q | Markedly reduced | ERT+ASMs+Ambroxol | ≥50% Reduction |
| Kim 2020-Patient 2 | N227S / R296Q | Markedly reduced | ERT+ASMs+Ambroxol | ≥50% Reduction |
| Kim 2020-Patient 3 | N227S / frameshift (V211fs) | Markedly reduced | ERT+ASMs+Ambroxol | ≥50% Reduction |
| Kim 2020-Patient 4 | F252I / L444P | Markedly reduced | ERT+ASMs+Ambroxol | ≥50% Reduction |
| Tonin 2018 | N188S / G82G | 1.9 (↓, ref 4.8–14 nmol/mg/h) | ERT+ASMs | No response |
| Miyahara 2009-Patient 4 | Not reported | Markedly reduced | ASMs | ≥50% Reduction |
| Seeman 1996 | V417L / T510I | Severely reduced (0.48% of normal) | ERT+ASMs | No response |

**Abbreviations: ASMs, antiseizure medications; BMT, Bone Marrow Transplantation; ERT, enzyme replacement therapy; PME, progressive myoclonus epilepsy; SRT, substrate reduction therapy.**
