# Supplementary figures and images for: Case Report: Progressive myoclonus epilepsy as an early manifestation of neuronopathic Gaucher disease
Source: Front Neurosci. 2026 Jan 27;20:1742318. doi: 10.3389/fnins.2026.1742318 (PMC12886469; doi:10.3389/fnins.2026.1742318)

(A)


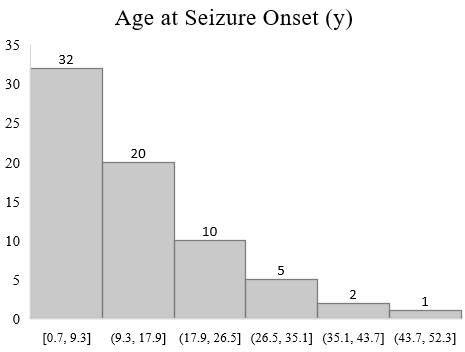


(B) (C)


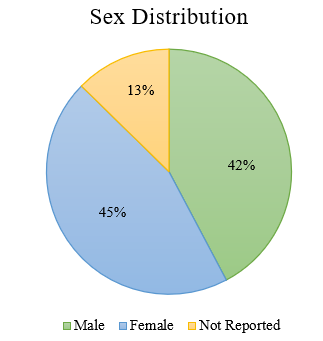

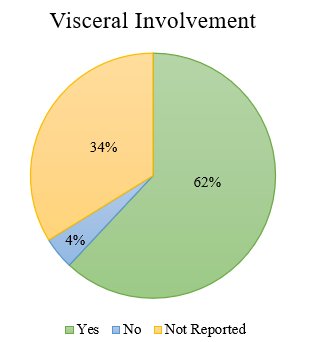


(D) (E)


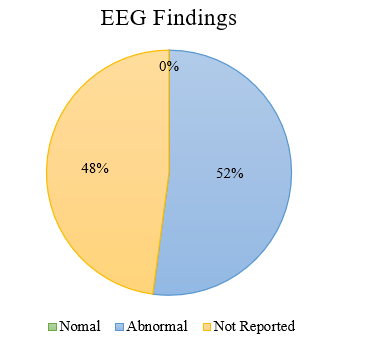

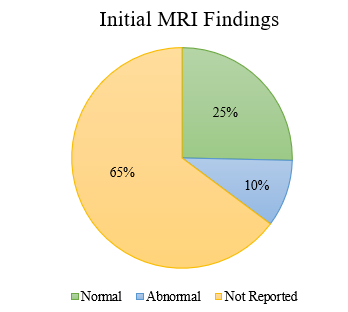


(F)


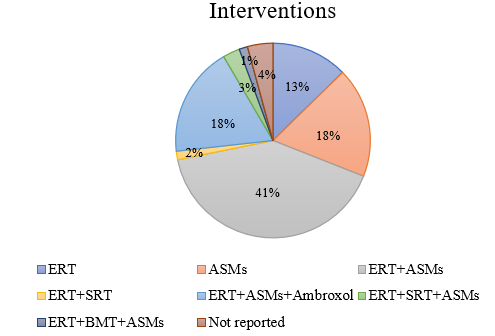


(G)


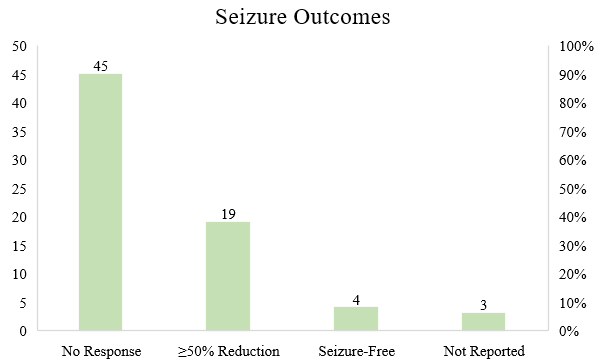

Supplement: Supplementary file 3 [file Table_3.DOCX]
